# Complimenting Dr. Kent

**Published:** 1889-01

**Authors:** Aspasia E. Ramborger

**Affiliations:** Secy.; Phila.


### COMPLIMENTING DR. KENT.

After the closing lecture of the Post-Graduate Course on Hahnemannian Philosophy and Materia Medica, delivered by Professor J. T. Kent, M. D., at the hospitals of the Woman's Homœopathic Association, Twentieth Street and Susquehanna Avenue, Philadelphia, Dr. G. B. Ehrmann was called to the chair.

The following resolutions were offered by Dr. A. G. Allan and unanimously accepted by the class of physicians and students:

WHEREAS, We the members of the first Post-Graduate Course of Hahnemannian Homœopathy known to the world, have kindly and faithfully received of Professor Kent the wonderful truth of Hahnemann's philosophy as given in the *Organon*, be it

*Resolved*, That we as a body tender our thanks to Professor Kent, wishing him long life, good health, and continued usefulness.

*Resolved*, That we offer him our hearty co-operation in all things to advance our common cause, Hahnemannian Homœopathy.

Dr. R. B. Johnstone offered the following resolutions, which were also adopted:

WHEREAS, The true homœopathists of Rochester, N. Y., as represented in the Hahnemannian Society of Rochester, have seen fit to sever their connection with the so-called Homœopathic Society for the reason that the said society is no longer homœopathic, but mongrel, and

WHEREAS, The said true homœopathic physicians have organized a true homœopathic society, called the Hahnemannian Society of Rochester, and are now engaged in an endeavor to erect a homœopathic hospital in that city, be it

*Resolved*, That we, the members of the Post-Graduate Course of Homœopathic Philosophy and Materia Medica, now just closing, do extend to the above true homœopathic physicians our most hearty sympathies and support. That a copy of these resolutions be sent to the Rochester Hahnemannian Society, the Central New York Homœopathic Medical Society, Rochester *Union and Advertiser*, *Homœopathic Physician*, *Medical Advance*, and the Philadelphia daily papers.

PHILA., Dec. 15th, 1888. ASPASIA E. RAMBORGER, *Secy.*

